# Medium-chain triglyceride supplementation in infants with biliary atresia: a review of the evidence for impacts on fat absorption, growth, nutritional status and clinical outcomes

**DOI:** 10.1017/S0029665125102140

**Published:** 2026-01-08

**Authors:** Sara Mancell, Anil Dhawan, Salma Ayis, Kevin Whelan

**Affiliations:** 1 Department of Nutritional Sciences, King’s College London, London, UK; 2 Department of Nutrition & Dietetics, King’s College Hospital NHS Foundation Trust, London, UK; 3 Paediatric Liver, GI and Nutrition, King’s College Hospital NHS Foundation Trust, London, UK; 4 Department of Population Health, King’s College London, London, UK

**Keywords:** Medium-chain triglyceride, MCT, infants, biliary atresia, cholestasis

## Abstract

Biliary atresia is a rare bile duct disease resulting in intestinal bile salt depletion due to poor bile flow. Medium-chain triglyceride (MCT) supplementation is widely recommended and used as the main dietary management in infants, however evidence for its use is limited and there is uncertainty regarding the optimal percentage (proportion of total fat that is MCT) and dose (grams/kilogram/day, g/kg/d). The aim was to review the evidence for the impact of MCT on fat absorption, growth, nutritional status and clinical outcomes in infants with biliary atresia and the optimal nutrition profile of MCT supplementation. A scoping review found that the mostly observational, historic evidence for MCT supplementation pointed to greater fat absorption during MCT supplementation compared to no supplementation, but also some evidence of a risk of essential fatty acid deficiency with very high MCT percentage. Only six studies have investigated MCT percentages and only three reported MCT dose. One analysis of MCT in the largest cohort of biliary atresia patients ever presented (n=200), found no association between MCT percentage with growth, nutritional status or clinical outcomes. Counterintuitively, there was an unexpected inverse association between MCT dose and growth. A possible interpretation was that increased MCT was a consequence of poor growth rather than a cause, as infants either drank more or dietitians prescribed more MCT as fat malabsorption worsened. In conclusion, MCT is widely recommended, however, the evidence for its use is lacking and there remains uncertainty about the optimum percentage and dose for infants with biliary atresia.

## Introduction

Biliary atresia is a rare congenital disease of the bile ducts occurring in one in 15,000-20,000 live births ^(1)^. A rapid inflammatory process causes bile duct fibrosis resulting in obstructed or absent bile ducts and a disruption of bile flow ^(2)^. When bile flow is disrupted the result is cholestasis, defined as the retention of biliary substances (bilirubin, bile salts, cholesterol) in the liver that are normally excreted into bile and eliminated in the small intestines ^(3, 4)^. As bile accumulates in the liver, bile salts overflow into the peripheral blood stream causing conjugated hyperbilirubinaemia and bile salts are then excreted in urine ^(5)^. Kasai portoenterostomy (KPE) surgery is performed as soon as possible (ideally within the first two months of life) to restore bile flow and prevent further hepatic damage ^(6, 7)^.

Bile flow may still be significantly reduced after KPE with serum total bilirubin remaining elevated ^(8)^. KPE is considered to have been successful if total bilirubin is ≤20 μmol/L within six months of the procedure ^(9)^, and when defined as such most studies report KPE success rates of over 50% ^(10, 11)^. In cases where bile flow is not successfully re-established (unsuccessful KPE) there is a high likelihood of liver transplant within the first year of life ^(9)^. However, even those who have a successful KPE are likely to develop end-stage liver disease and require a liver transplant due to the progressive, destructive disease process characteristic of biliary atresia ^(9)^. The result is that half of infants with biliary atresia require a liver transplant by the age of two years and 75% by the age of 20 years ^(12)^.

**Figure 1:**
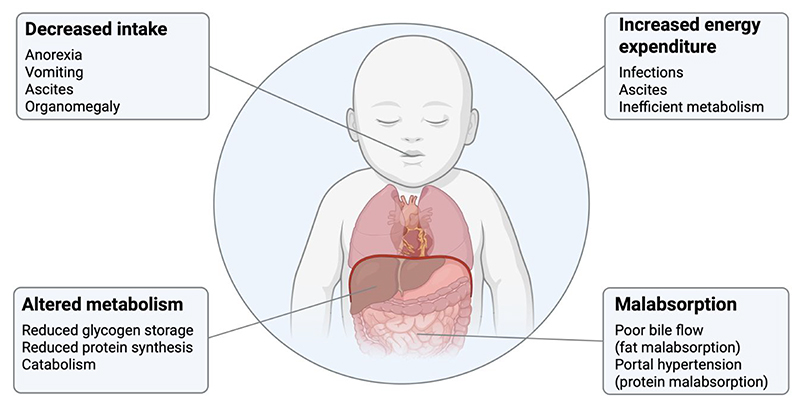
Pathophysiology of malnutrition in biliary atresia. Factors contributing to malnutrition are shown and include decreased intake, altered metabolism, increased energy expenditure and malabsorption. Created in BioRender by S. Mancell (2025), https://BioRender.com/meca5pe

The nutritional consequences of biliary atresia associated with poor bile flow include fat malabsorption, faltering growth and poor nutritional status. As liver disease progresses towards end-stage liver failure the nutritional consequences may additionally include poor oral intake, altered nutrient malabsorption and increased energy expenditure which can increase the risk of malnutrition, worsen growth failure and have a detrimental impact on clinical outcomes ^(13)^. The pathophysiology of malnutrition in biliary atresia is shown in Figure 1. Fat malabsorption results because bile is unable to flow to the intestines resulting in a depletion of small intestinal bile salts which are required for the digestion of fat, specifically long-chain triglycerides (LCTs) ^(14)^. The extent of fat malabsorption in biliary atresia is not known. The coefficient of fat absorption (percentage of total fat absorbed) as measured through stool fat balance studies has been reported to be anywhere from 23% to 84% in children with cholestatic liver disease ^(15–19)^. Fat provides a significant amount of energy in the infant diet as it comprises up to half of the energy in breastmilk and infant formula milks ^(20)^. Fat malabsorption therefore increases the risk of faltering growth in infants with biliary atresia. In addition to fat malabsorption as a cause of malnutrition in biliary atresia, complications of end-stage liver disease may also contribute. These include decreased intake due to anorexia, vomiting, ascites and organomegaly; protein malabsorption due to portal hypertension; altered metabolism resulting from reduced glycogen storage, reduced protein synthesis and catabolism; and increased energy expenditure due to infections, ascites and metabolic changes ^(13, 21)^.

### Medium-chain triglycerides

Dietary management of fat malabsorption in biliary atresia involves supplementation with medium-chain triglycerides (MCT). MCTs were first introduced clinically in the 1950s as an energy substitute for LCT in lipid malabsorption disorders ^(22)^. They have been used in a wide variety of conditions including pancreatic insufficiency, chylous ascites, chylothorax, intestinal failure, epilepsy ^(23–25)^, long-chain fatty acid oxidation disorders ^(26)^ and in premature infants ^(20)^. The structure, absorption and metabolism of MCTs make them potentially useful in biliary atresia and other cholestatic liver diseases.

**Figure 2:**
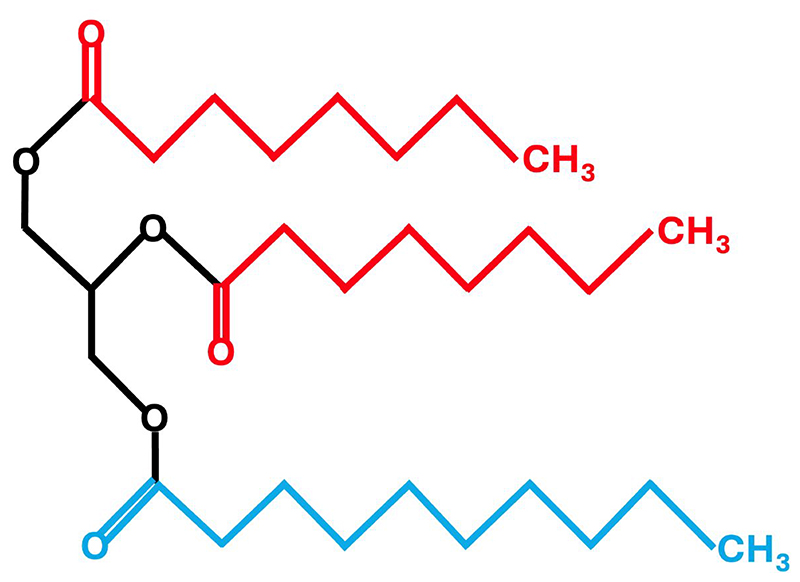
Chemical structure of a medium-chain triglyceride consisting of three medium-chain fatty acids. Caprylic acid (C8) is shown in red and capric acid (C10) is shown in blue.

Triglycerides, comprised of a glycerol backbone and three fatty acids, are classified based on the length of the fatty acids as short (2-6 carbons), medium (8-12 carbons) or long (≥14 carbons) by the FAO/WHO Expert Consultation ^(27)^. However, this consultation recognised that this definition is not universal and there are various definitions in the literature. For example, caproic acid (C6) is classified in some reviews as a medium-chain fatty acid (MCFA) ^(23, 24, 28, 29)^ and lauric acid (C12) has been classified as a long-chain fatty acid (LCFA) ^(29)^. Medium-chain triglycerides are comprised primarily of the MCFAs caprylic (C8; 50-80%) and capric (C10; 20-50%) fatty acids and a very small amount (2-4%) of caproic (C6) and lauric (C12) fatty acids ^(22)^. Unlike LCTs, MCTs do not contain the long-chain essential fatty acids, linoleic (C18:2) and alpha-linolenic (C18:3) acids and are therefore unable to serve as a precursor to the synthesis of eicosanoids, docosahexaenoic acid (C22:6) and eicosapentaenoic acid (C20:5) ^(24)^. Compared to LCTs, MCTs are partially water-soluble, have a lower smoke point, smaller molecular weight, are liquid at room temperature and are less energy dense (8.4 versus 9.2 kcal/g) ^(23)^. An example of the chemical structure of an MCT is shown in Figure 2.

**Figure 3:**
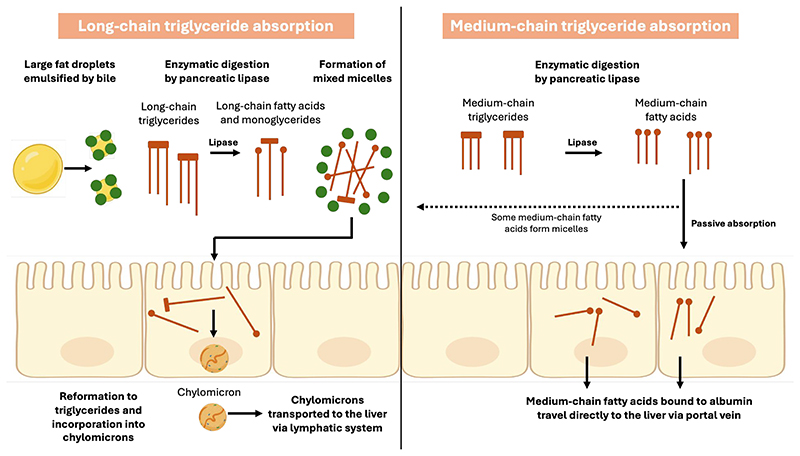
Absorption of long-chain and medium-chain triglycerides. Long-chain fatty acids are incorporated into micelles and then into chylomicrons for transport via the lymphatic system, whereas medium-chain fatty acids are passively absorbed and travel directly to the liver via the portal vein. Created in BioRender by S Mancell (2025), https://BioRender.com/ta609tx

In addition to structural differences, MCTs also differ from LCTs in the way in which they are absorbed and transported from the small intestine to the liver as shown in Figure 3. Medium-chain triglycerides are partially hydrolysed in the stomach by pre-duodenal lipases in the same way as LCTs although for MCTs hydrolysis may happen at a faster rate ^(30)^. An *in vitro* study using gastric aspirates from healthy newborn infants showed that hydrolysis of MCTs was 5-8 times faster than LCTs ^(31)^ and gastric emptying of MCTs has also been shown to be faster ^(25)^. Studies have shown that MCTs either do not stimulate cholecystokinin ^(25)^ or may stimulate cholecystokinin to a lesser degree than LCT ^(23)^ and may be less reliant on pancreatic lipase for their hydrolysis ^(25)^. In a small crossover study of six adults with pancreatic insufficiency supplemented with MCT or LCT for five days, steatorrhoea was significantly reduced during MCT supplementation compared to LCT due to reduced reliance on pancreatic lipase for absorption in the former ^(32)^.

Unlike LCTs, the majority of MCTs do not require bile for emulsification and therefore do not form mixed micelles for absorption across enterocytes and are not incorporated into chylomicrons for transport via the lymphatic system to the liver ^(23, 33)^. Instead, the majority of MCFAs are passively absorbed and are transported directly to the liver via the portal vein bound to albumin ^(22, 34)^. However, there are some MCFAs that are absorbed in the same way as LCFAs and this may be dependent on fatty acid chain length with longer MCFAs (C10, C12) more likely to be transported in the same way as described for LCFAs ^(23, 35)^. In a small study of four adults receiving MCT supplementation for six days, there were three to four times more C10 incorporated into triglycerides despite supplementation providing twice as many C8 fatty acids ^(36)^. The authors suggested that this demonstrated a preference for MCFAs with longer chain lengths to form chylomicron triglycerides when MCT supplementation was administered ^(36)^. This study was undertaken in healthy adults. No studies were identified that investigated the proportion of MCFAs incorporated into triglycerides when MCT supplementation is provided to children with cholestatic liver disease. In the liver, MCFAs are absorbed directly into hepatocytes and enter mitochondria independently of carnitine, unlike LCFAs ^(35)^. As a result, MCFA are more available for oxidation than re-esterification into triglycerides whereas the majority of LCFAs are incorporated into triglycerides ^(37)^. MCFAs are therefore metabolised at a higher rate than LCFAs and are a more rapid source of energy rather than being used for fat deposition ^(23, 28)^. This is thought to explain why dietary induced thermogenesis has been shown to be higher during MCT supplementation compared to no MCT ^(38)^.

Premature infants may benefit from MCT supplementation as they have been shown to have limited pancreatic lipase and bile salts ^(20)^. The impact of MCT percentage on short-term growth in premature infants was the subject of a recent Cochrane review that identified 10 trials (nine randomised and one quasi-randomised) including 253 infants ^(20)^. The review found no difference in growth between those receiving <30% MCT compared to >30% MCT formula milks with a mean difference in weight of 0.0 g (95% CI -5.93, 5.93), length 0.10 cm/week (95% CI -0.09, 0.29) and head circumference -0.04 cm/week (95% CI -0.17, 0.09) ^(20)^. The authors concluded that the evidence for an association between MCT percentage and short-term growth was limited and of low to very low certainty ^(20)^.

### Sources of medium-chain triglycerides

The majority of dietary fats are LCTs with only very small amounts of MCTs occurring naturally ^(23)^. The main sources of MCT are coconut oil and palm kernel oil that contain over 50% MCT ^(23, 39, 40)^. Cow’s milk is a source of MCT with 6-17% of fatty acids being MCFAs ^(41)^. MCTs are also found in human milk primarily incorporated into triglycerides with a single MCFA alongside two LCFAs ^(42)^, with MCFAs constituting between 2% and 10% of all fatty acids in human milk ^(43, 44)^. Data regarding the MCFA composition of coconut oil, palm kernel oil, cow’s milk and human milk has been synthesized into Table 1. Medium-chain triglycerides may be incorporated into specialist commercial formula milks (“MCT formula milk”) or added manually to breastmilk, standard formula milk or MCT formula milk as an oil or emulsion. Commercial MCT formulations may contain naturally-derived MCT oil or synthetic MCT oil (where MCFA are hydrolysed from coconut or palm kernel oil and then re-esterified onto a glycerol backbone) ^(45)^.

### Medium-chain triglyceride supplementation in biliary atresia

The rationale for MCT supplementation in biliary atresia where intestinal bile salts are absent or depleted is that providing fat that can be absorbed, even if it is lower in energy than LCT and may be less likely to contribute to fat stores, should result in increased available energy for absorption, therefore helping to facilitate improved growth and nutritional status and ideally improve clinical outcomes. Extensive practice and clinical guidelines support the use of MCT supplementation in liver disease ^(21, 46, 47)^. Despite the important potential mechanisms of action and widely recommended guidelines to support practice, there was previously no systematic assimilation of research in the area of MCT supplementation in biliary atresia. Recently, a scoping review identified 24 studies investigating associations and impacts of MCT in children with cholestatic liver disease, 20 of which included infants with biliary atresia ^(48)^. There were three RCTs (including 19 infants), one non-randomised controlled trial, seven uncontrolled trials and 13 case reports and series. The review demonstrated that the mostly observational evidence for MCT supplementation is limited both in terms of quantity and quality and most studies were published more than 30 years ago ^(48)^. A summary of studies of MCT in biliary atresia is provided below.

#### Evidence for impact on fat absorption

Although MCT supplementation is recommended to replace the energy lost due to fat malabsorption, the evidence for the impact of MCT on fat absorption is limited. As part of the recent scoping review mentioned above ^(48)^, 10 studies were identified that measured fat absorption in 87 children with cholestatic liver disease (the majority of whom had biliary atresia). There was one RCT including two children, one non-randomised controlled trial, six uncontrolled trials and two case series. Fat absorption was measured and reported in different ways making comparison difficult, however all nine studies reported that fat absorption was greater during MCT supplementation compared to no MCT supplementation ^(15–19, 49–52)^. Fat absorption during MCT supplementation was reported to be 73-100% whereas during no MCT supplementation fat absorption was 20-97% ^(48)^. In the one study that compared fat absorption during supplementation with similar MCT percentages (42% and 48%) no differences in absorption were reported ^(53)^. Overall, the limited evidence points towards greater absorption of fat during MCT supplementation compared to no MCT, but with limited quality trials and therefore limited certainty.

#### Evidence for impact on growth

The aim of MCT supplementation in terms of growth is to prevent or reduce growth failure related to fat malabsorption. The recent scoping review identified 14 studies that measured growth, although four of the studies only included qualitative statements related to growth rather than actual growth data and growth was measured over different periods ranging from three days to one year ^(48)^. A further challenge was that growth was reported in a variety of ways such as kilograms (kg) or kg/month with only six studies presenting z-scores. In the four studies that compared MCT with no MCT, growth was greater during MCT supplementation compared to no MCT in only nine out of a total of 58 children and there were no differences in growth in the other children ^(16, 18, 49, 51)^. In the studies comparing MCT percentages, there was no difference in growth in three studies including only 30 children ^(53–55)^ while in one conference abstract including nine children growth was greater when low and high percentage MCT were provided compared to medium percentage MCT ^(56)^. The limited evidence from the scoping review in relation to the impact of MCT supplementation was unclear with no strong evidence of an association between MCT supplementation and growth ^(48)^.

**Figure 4:**
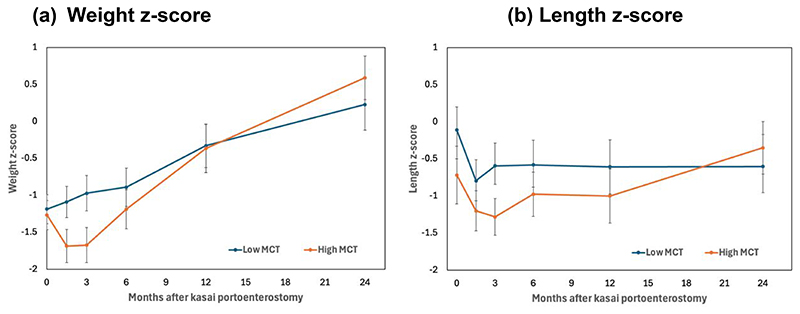
Growth following Kasai portoenterostomy by MCT amount (g/kg/d). a) Mean weight z-score and (b) mean length z-score plus 95% confidence intervals in the two years post Kasai portoenterostomy are shown for those having low MCT (<2.7g/kg/d MCT) versus high MCT (≥2.7 g/kg/d MCT) ^(57)^.

The association between MCT supplementation and growth was investigated further in a recent retrospective review which described MCT supplementation and investigated its association with a range of outcomes in the largest cohort of biliary atresia patients ever presented (n=200) ^(57)^. Growth measurements (weight, length, head circumference and mid-upper arm circumference) were reported as raw measurements, z-scores and change in z-score from baseline in the two years post Kasai portoenterostomy. No association was found between MCT (percentage or g/kg/d) and growth beyond three months. There was, however, an unexpected inverse association between MCT (g/kg/d) with growth in the two years after diagnosis as mixed model analysis demonstrated a rate of change in weight z-score of -0.27 (95% CI -0.37 to -0.17) per unit MCT (g/kg/d) (p<0.001) ^(57)^. A cautious interpretation (given that this was an observational study) was that increased MCT supplementation was a consequence of poor growth rather than a cause, as infants either drank more or dietitians prescribed more MCT as fat malabsorption worsened. There were no differences in growth based on MCT amount (g/kg/d) beyond three months as shown in Figure 4 and therefore the high MCT intake could potentially have facilitated infants to catch up to those consuming a lower intake of MCT who may have had less malabsorption initially ^(57)^.

#### Evidence for impact on biochemical nutritional status

Given that MCT is not a source of essential fatty acids and both LCT and intestinal bile salts are required to absorb fat-soluble vitamins and essential fatty acids, guidelines are that infants with biliary atresia should be provided with both MCT and LCT although the ideal proportions of each are not known ^(21, 46)^. The scoping review identified four studies in only 10 children indicating an association between very high percentage MCT (≥80%) and essential fatty acid deficiency ^(8, 54, 58, 59)^, although the conclusion from the scoping review was that the risk of deficiency may have been related to low LCT intake and malabsorption of LCT rather than to the MCT percentage of the formula milk *per se* ^(48)^. For example, previous studies have shown that children with cholestatic liver disease having LCT (with no MCT supplementation) still had significantly depleted plasma essential fatty acids ^(60, 61)^. Current guidelines are for infants with cholestasis to have 3% energy from linoleic acid and 0.7-1% from alpha-linolenic acid to avoid deficiency ^(21)^.

The scoping review did not identify any studies that investigated the association between MCT supplementation and fat-soluble vitamin deficiency ^(48)^. The retrospective review, the first study to investigate this, found no overall association between MCT (percentage or g/kg/d) with fat-soluble vitamin concentrations ^(57)^. At very high amounts of MCT supplementation (2-4 g/kg/d MCT) there was an inverse association between MCT and fat-soluble vitamin concentrations in the two years post Kasai portoenterostomy with a rate of change of -7.29 (95% CI -12.54, -2.05) nmol/L vitamin D and -3.44 (-4.98, -1.89) µmol/L vitamin E per unit MCT (g/kg/d) (p<0.01 for both). This finding was thought to support the theory that increased MCT supplementation was a consequence of poor growth and fat malabsorption (and therefore fat-soluble vitamin malabsorption) rather than a cause ^(57)^.

#### Evidence for impact on clinical outcomes

While there is good evidence of an association between growth and clinical outcomes ^(62–64)^, no studies were identified as part of the scoping review that investigated the association between MCT supplementation and clinical outcomes ^(48)^. Meanwhile, the retrospective review found no association between MCT supplementation with any clinical outcomes including cholangitis, gastrointestinal bleeding, ascites, liver-related blood tests, Kasai portoenterostomy success and two-year outcome defined as good (alive with native liver) or poor (received a liver transplant/died) ^(57)^. There were, however, several variables that were associated with poor growth including gastrointestinal bleeding, raised total bilirubin and a poor two-year outcome ^(57)^.

### Research and practice challenges

#### MCT percentage vs MCT dose

The amount of MCT provided to infants can be presented either as the MCT percentage (proportion of total fat that is MCT) or the MCT dose (MCT grams per kilogram per day (g/kg/d)). For infants receiving MCT formula milk only, the amount of MCT (percentage, dose) would be determined based on manufacturer information. For infants additionally provided with breastmilk, MCT percentage would be lower than for MCT formula milk alone given that breastmilk primarily provides LCT with only a small amount of MCT (Table 1) and it would not be possible to determine MCT percentage from both formula and breastmilk unless breastmilk intake itself were measured. Presenting MCT amount in g/kg/d would not take account of the small amount of MCT derived from breastmilk (unless breastmilk intake were measured) but would reflect the MCT dose from MCT supplementation.

#### Clinical guidelines and recommendations for MCT supplementation

The amount of MCT provided to infants has been defined using standard cutoffs: low (<40% MCT), medium (40-59% MCT), high (60-79% MCT) or very high (≥80% MCT) ^(48)^. However, there is no consensus on the ideal percentage to provide to infants with biliary atresia and only six studies so far have investigated MCT percentage ^(53–57, 65)^. Clinical guidelines make widely varied recommendations for MCT supplementation in infants with biliary atresia, likely due to the limited evidence base. For example, guidelines from Europe and North America (ESPGHAN, NASPGHAN) recommend 30% MCT with adjustments based on growth ^(21)^, from India (INASL) recommend 30-50% MCT ^(66)^, from Mexico (AMH) recommend 30-70% MCT ^(67)^, while clinical reviews recommend 50% MCT ^(47)^, 30-60% ^(68)^, 30-70% MCT ^(46, 47)^ and up to 75% MCT ^(69)^. In the retrospective review, the majority of infants had medium MCT percentage (n=129) or high MCT percentage (n=62) formula milk following Kasai portoenterostomy, however this was just from one centre ^(57)^. There are no published surveys of practices across any one country.

In terms of dose of MCT (g/kg/d), there is also no evidenced-based consensus on the ideal amount to provide to infants with biliary atresia and only three studies in the literature have reported intake in g/kg/d ^(19, 50, 57)^. One clinical guideline from India (IAP) ^(70)^ and several clinical reviews recommended 1-2 mL/kg/d MCT ^(12, 68, 71–73)^ and in some cases specified that the MCT dose should be divided into two to four doses during the day ^(12, 70–73)^. One review recommended 0.3 g/kg/d MCT ^(74)^ while another recommendation was to use a 4% MCT emulsion (providing 4 mL of product or 2 mL of MCT for every 100ml of formula milk) ^(47)^. Recommendations for MCT amount (percentage, dose) from clinical guidelines and reviews are synthesised in Table 2.

#### MCT percentage depends on total fat content

A challenge related to classifying MCT formula milks according to their MCT percentage is that higher MCT percentage formula milks may actually contain a lower MCT dose than lower MCT percentage formula milks due to differences in total fat. Table 3 shows selected examples of four MCT formula milks currently available in the United Kingdom. The example medium percentage MCT (50% of total fat) formula milk shown contains 2.8 g MCT whereas the example very high percentage MCT, (84% of total fat) contains only 2.5 g MCT per 100 kcal, due to a lower amount of total fat in the latter. As MCT percentage may be misleading in terms of actual MCT dose, reporting MCT g/kg/d as well as MCT percentage is recommended in the research setting ^(48)^ and may be helpful in clinical practice.

#### MCT supplementation and breastfeeding

The retrospective review found that breastfeeding did not appear to be associated with growth, nutritional status or clinical outcomes although infants who received breastmilk consumed less MCT than those who did not, a novel but unsurprising finding ^(57)^. A recent prospective observational study of 447 infants with biliary atresia found that receiving breastmilk was associated with increased weight gain and a lower total bilirubin at three months compared to those receiving formula milk only ^(75)^. Formula milks in this study included standard, soy, hydrolysed, amino acid and MCT formula milks, however volume and concentration of formula milks were not reported ^(75)^. In view of the significant advantages of breastmilk, for example in terms of immunity, gut function, obesity and cognitive function ^(76)^ and the potential for improved weight gain, it would seem sensible to recommend breastfeeding alongside MCT supplementation although more research is needed to determine the impact of breastfeeding on outcomes and the type and amount of MCT supplementation to recommend.

#### Type of MCT supplementation

There is little evidence for the ideal type of MCT supplementation to provide (i.e. MCT formula milk, emulsion or oil). Some reviews have specified that MCT formula milk should only be provided to infants with cholestasis if breastfeeding is not available ^(21, 77, 78)^ while other recommendations are to provide MCT formula milk alongside breastfeeding ^(47)^. Recommendations include adding MCT as an oil or emulsion to MCT formula milk ^(47, 73)^, expressed breast milk ^(70, 71)^ or standard infant formula milk ^(79)^. Our retrospective review showed that the majority of infants received MCT formula milk either alone or alongside breastfeeding and very few received MCT as an oil or emulsion alongside breastfeeding ^(57)^.

A challenge of administering MCT as a separate oil or emulsion to breastmilk or a standard formula milk in clinical practice is that this reduces the protein to energy ratio. The optimal protein to energy ratio for infants with biliary atresia is not known, however WHO guidelines for optimal catch-up growth (10-20 g/kg/d) recommend a protein to energy ratio of 8.9-11.5% ^(80)^. The extent of fat malabsorption in biliary atresia is not known, however it is likely that the amount of energy absorbed from LCT is lower than what is consumed, and this has implications for the protein to energy ratio. There may be an argument for providing both protein and MCT supplementation as this would provide a more favourable protein to energy ratio and allow the maximum amount of breastfeeding alongside MCT supplementation. More research is required to determine the ideal protein to energy ratio and the extent of malabsorption in infants with biliary atresia.

## Conclusion

This review critically reviewed the evidence for how, if at all, MCT supplementation impacts on fat absorption, growth, nutritional status and clinical outcomes in biliary atresia and the optimal nutrition profile of MCT supplements and formula milks. The evidence for MCT supplementation is limited both in terms of quantity and quality but does point towards improved fat absorption and a risk of essential fatty acid deficiency during supplementation with very high MCT percentage formula milks that lack an LCT source (for example infants who do not receive breastfeeding). Infants with fat malabsorption and poor growth early after Kasai portoenterostomy may consume high amounts of MCT (g/kg/d) to compensate and it is possible that this may help them to achieve catch-up weight gain. More research is required on the impact of breastfeeding on outcomes and the type of MCT supplementation that should be provided alongside breastfeeding. There is insufficient evidence currently to recommend an ideal MCT amount (percentage or g/kg/d) for infants with biliary atresia. High quality RCTs are now required to investigate MCT supplementation in biliary atresia and inform clinical decision-making. Clinical trials of nutrition interventions in infants with biliary atresia are challenging, firstly due to low numbers in view of the rare disease ^(81)^ and the fact that recruitment to trials can be challenging at a time of great stress for parents ^(82)^. Methods to overcome these challenges, including multi-centre trials and novel approaches to supporting and informing parents ^(83)^ are required.

## Figures and Tables

**Table 1:** Medium-chain fatty acid content of coconut oil, palm kernel oil, cow’s milk and human milk as a percentage of total fatty acids

Medium-chain fatty acids	Percentage of total fatty acids			
	Coconut oil ^(39)^	Palm kernel oil ^(40)^	Cow’s milk ^(41)^	Human milk ^(43)^
Caproic (C6)	0.7	0.1	2.4	0.0
Caprylic (C8)	8.6	2.9	1.4	0.0
Capric (C10)	6.3	3.0	2.7	0.7
Lauric (C12)	47.6	45.6	3.3	3.4

**Table 2:** Summary of contrasting recommendations for MCT percentage and MCT dose in biliary atresia taken from clinical guidelines and reviews

MCT percentage or dose recommended	Evidence type	Year of publication	Reference
30%	Clinical Guideline from Europe (ESPGHAN) and North America (NASPGHAN)	2019	^(21)^
30-50%	Clinical Guideline from India (INASL)	2021	^(66)^
30-70%	Clinical Guideline from Mexico (AMH)	2022	^(67)^
50%	Clinical review	2011	^(47)^
30-60%	Clinical review	2022	^(68)^
30-70%	Clinical review	2007, 2011	^(46, 47)^
≤ 75%	Clinical review	2017	^(69)^
1-2 mL/kg/d	Clinical Guideline from India (IAP)	2014	^(70)^
1-2 mL/kg/d	Clinical review	2011-2022	^(12, 68, 71–73)^
0.3 g/kg/d	Clinical review	2002	^(74)^
2 mL per 100mL formula milk	Clinical review	2011	^(47)^

MCT, medium-chain triglyceride; ESPGHAN, European Society for Pediatric Gastroenterology Hepatology and Nutrition; NASPGHAN, North American Society for Pediatric Gastroenterology Hepatology and Nutrition; INASL, Indian National Association for Study of the Liver; AMH: Asociación Mexicana de Hepatología; IAP, Indian Academy of Pediatrics.

**Table 3:** Selected examples of MCT formula milks per 100 kcal available in the United Kingdom

MCT Percentage classification ^(48)^	Energy kcal	MCT %	Total fat g	MCT dose g
Example of a low MCT % (<40%)	100	33	5.4	1.8
Example of a medium MCT % (40-59%)	100	50	5.4	2.8
Example of a high MCT % (60-79%)	100	60	3.8	2.0
Example of a very high MCT % (≥80%)	100	84	3.0	2.5

MCT: medium-chain triglyceride.
